# EXPECTATIONS OF RECOGNITION AND BENEFITS OF CREATIVE ACTIVITIES FOR MIDDLE AND OLDER ADULTS

**DOI:** 10.1093/geroni/igac059.2613

**Published:** 2022-12-20

**Authors:** Darby Mackenstadt, Carolyn Adams-Price, Sarah Israel

**Affiliations:** Mississippi State University, Starkville, Mississippi, United States; Mississippi State University, STARKVILLE, Mississippi, United States; Mississippi State University, Starkville, Mississippi, United States

## Abstract

Creative hobbies have been found to be beneficial for mental health, especially if an individual considers their hobby an important part of their identity (Adams-Price & Morse, 2018). Although receiving recognition from friends or family for one’s creative abilities has been seen to decrease depressive symptoms for adults, these benefits may depend on expectations and type of recognition desired (Israel et al, 2020). Those who participate in creative activities as a career compared to those who participate as hobbyist may not receive the same benefits due to the stress artists endure trying to earn a living (Barker et al, 2009). This study aimed to explore the relationships between hobby recognition, hobby’s impact on identity, and mental well-being. A sample of 279 primarily white (88%) female (95.3%) adults aged 40 to 84 (M = 59.9-years-old) were interviewed during the Covid-19 Pandemic. A structural equation model was developed in AMOS 28.0 to correlate these variables and do a multiple groups analysis, comparing 100 older adults (65+) and 179 middle aged adults (40-64) who all reported at least one creative hobby. Results found a negative relationship between identifying with a creative hobby and receiving recognition for the hobby, a negative relationship between mental well-being and recognition, and a positive relationship between identifying with one’s hobby and mental well-being. The results suggest that recognition from others may have mixed effects on mental health but identifying with one’s creative hobby may be a protective factor. Implications expectations of recognition will be discussed.

